# Disordering of the vortex lattice through successive destruction of positional and orientational order in a weakly pinned Co_0.0075_NbSe_2_ single crystal

**DOI:** 10.1038/srep10613

**Published:** 2015-06-03

**Authors:** Somesh Chandra Ganguli, Harkirat Singh, Garima Saraswat, Rini Ganguly, Vivas Bagwe, Parasharam Shirage, Arumugam Thamizhavel, Pratap Raychaudhuri

**Affiliations:** 1 Tata Institute of Fundamental Research, Homi Bhabha Road, Colaba, Mumbai 400005, India; 2 Indian Institute of Technology Indore, IET-DAVV Campus, Khandwa Road, Indore 452017, India

## Abstract

The vortex lattice in a Type II superconductor provides a versatile model system to investigate the order-disorder transition in a periodic medium in the presence of random pinning. Here, using scanning tunnelling spectroscopy in a weakly pinned Co_0.0075_NbSe_2_ single crystal, we show that the vortex lattice in a 3-dimensional superconductor disorders through successive destruction of positional and orientational order, as the magnetic field is increased across the peak effect. At the onset of the peak effect, the equilibrium quasi-long range ordered state transforms into an orientational glass through the proliferation of dislocations. At a higher field, the dislocations dissociate into isolated disclination giving rise to an amorphous vortex glass. We also show the existence of a variety of additional non-equilibrium metastable states, which can be accessed through different thermomagnetic cycling.

Understanding the evolution of the structure of the vortex lattice (VL) in a weakly pinned type II superconductor is of paramount importance since it determines superconducting properties that are directly relevant for applications, i.e. critical current and the onset of electrical resistance. Over the past two decades, there have been intense efforts to understand the nature of the order-disorder transition of the VL with temperature or magnetic field^1^,^2^,^3^. It is generally accepted that in a clean system the hexagonal VL realised at low temperature and magnetic field, can transform to vortex liquid above a characteristic temperature (*T*) and magnetic field (*H*). Random pinning, arising from crystalline imperfection in the superconductor significantly complicates this scenario. It has been argued that since the system can no longer sustain true long-range order, both the ordered and the disordered state can become of glassy nature^4^,^5^, characterised by different degree of positional and orientational order. In addition, the VL can exist in a variety of non-equilibrium metastable states^6^,^7^, depending on the thermomagnetic history of the sample.

In contrast to the VL in superconducting thin films, where the order-disorder transition can be understood within the framework of Berezinski-Kosterlitz-Thouless-Halperin-Nelson-Young theory of 2-dimensional (2-D) melting^8^,^9^,^10^, the VL in a 3-dimensional (3-D) superconductor presents a more challenging problem. In this case the vortex line is rigid only up to a length scale much shorter than sample dimensions. Thus in a weakly pinned single crystal, the vortex line can bend considerably along the length of the vortex. It is generally accepted that in the presence of weak pinning the Abrikosov VL can transform into a quasi long-range ordered state such as Bragg glass^11^ (BG), which retains long-range orientational order of a perfect hexagonal lattice but where the positional order decays algebraically with distance. Theoretically, both the possibility of a direct first order transition from a BG to a vortex glass (VG) state^12^,^13^ (with short range positional and orientational order) as well as transitions through an intermediate state, such as multi-domain glass or a hexatic glass^14^,^15^, have been discussed in the literature. While many experiments find evidence of a first-order order-disorder transition^16^,^17^,^18^,^19^, additional continuous transitions and crossovers have been reported in other regions^20^,^21^,^22^ of the *H-T* parameter space, both in low-*T*_*c*_ conventional superconductors and in layered high-*T*_*c*_ cuprates.

Experimentally, the order-disorder transition in 3-D superconductors has been extensively studied through bulk measurements, such as critical current^23^, ac susceptibility^24^,^25^ and dc magnetisation^26^,^27^. These studies rely on the fact that in the presence of random pinning centres, the VL gets more strongly pinned to the crystal lattice as the perfect hexagonal order of the VL is relaxed^28^. The order-disorder transition thus manifests as sudden non-monotonic enhancement of bulk pinning^29^, and consequently of the critical current and the diamagnetic response in ac susceptibility measurements. Known as the “peak effect”, this has been a central theme of many studies on the static and dynamic properties of VLs. These measurements, although valuable in establishing the phase diagram of type II superconductors, do not reveal the evolution of the microscopic structure of the VL across the order-disorder transition. A more direct, though less used method, is through direct imaging of the VL using scanning tunnelling spectroscopy^30^,^31^,^32^,^33^,^34^ (STS). The main challenge in this technique is to get large area images that are representative of the VL in the bulk crystal.

Here, we track the evolution of the equilibrium state of the VL across the magnetic field driven peak effect at low temperature using direct imaging of the VL using STS in an NbSe_2_ single crystal, intercalated with 0.75% of Co. It has been shown^35^ that the intercalated Co atoms have two competing effects. On the one hand, the intercalated Co atoms generate random pinning centres for the vortices. On the other hand, the anisotropy in the upper critical field reduces compared to undoped NbSe_2_, thereby making the vortex lines ( for *H*||*c* ) stiffer and hence less adaptable to bending to accommodate the point pinning centers of Co. Consequently, in weakly Co intercalated NbSe_2_ single crystals the VL lattice becomes very weakly pinned as manifested by nearly vanishing critical current^35^ at low magnetic fields and a pronounced peak effect close to *H*_*c2*_. We analyse VL images consisting of several hundred to a thousand vortices at 350 mK, taken across the magnetic field driven the peak effect. For low fields, the stable state of the VL has nearly perfect hexagonal structure, with long range orientational order and a slowly decaying positional order. At the onset of the peak effect, dislocations proliferate in the VL, transforming the VL to an orientational glass (OG) with slowly decaying orientational order. Above the peak of the peak effect, dislocations dissociate into isolated disclinations driving the VL into an amorphous vortex glass (VG) which connects smoothly to the liquid state close to the upper critical field, *H*_*c2*_.

## Results

### Bulk pinning properties

**Figure 1:**
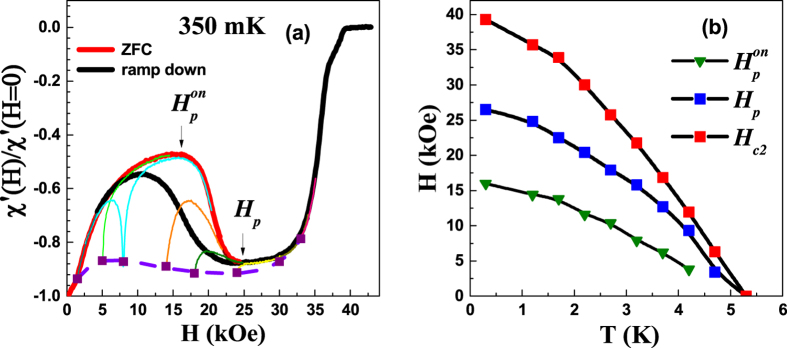
(**a**) Magnetic field (*H*) dependence of the real part of linear ac susceptibility (χ’) (normalised to its value in zero field) at 350 mK for the VL prepared using different thermomagnetic cycling. The red line is χ’-*H* when the magnetic field is slowly ramped up after cooling the sample in zero field (ZFC state). The black line is χ’-*H* when the magnetic field is ramped down from a value higher than *H*_*c2*_. The square symbols stand for the χ’ for the FC states obtained by cooling the sample from *T* *>* *T*_*c*_in the corresponding field; the dashed line shows the locus of these FC states created at different *H.* The thin lines starting from the square symbols show the evolution of χ’ when the magnetic field is ramped up or ramped down (ramped down segment shown only for 0.8 T), after preparing the VL in the FC state. We observe that the FC state is extremely unstable to any perturbation in magnetic field. χ’ is normalised to the zero field value for the ZFC state. (**b**) Phase diagram showing the temperature evolution of 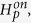
*H*_*p*_ and *H*_*c2*_ as a function of temperature.

Figure 1(a) shows the bulk pinning response of the VL at 350 mK, measured from the real part of the linear ac susceptibility (χ’) (see Supplementary Information) when the sample is cycled through different thermomagnetic histories. The χ’-*H* for the zero field cooled (ZFC) state (red line) is obtained while ramping up the magnetic field after cooling the sample to 350 mK in zero magnetic field. The “peak effect” manifests as a sudden increase in the diamagnetic response between 16 kOe (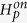) to 25 kOe (*H*_*p*_) after which χ’ monotonically increases up to *H*_*c2*_ ~ 38 kOe. When the magnetic field is ramped down after reaching a value *H* *>* *H*_*c2*_ (black line, henceforth referred as the ramp down branch), we observe a hysteresis starting below *H*_*p*_ and extending well below 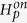. A much more disordered state of the VL with stronger diamagnetic response is obtained when the sample is field cooled (FC), by applying a field at 7 K and cooling the sample to 350 mK in the presence of the field (solid squares). This is however a non-equilibrium state: When the magnetic field is ramped up or ramped down from the pristine FC state, χ’ merges with the ZFC branch or the ramp down branch respectively. In contrast, χ’ for the ZFC state is reversible with magnetic field cycling up to 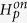, suggesting that it is the more stable state of the system. Figure 1(b) shows the phase diagram with 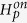, *H*_*p*_ and *H*_*c2*_, obtained from isothermal χ’-*H* scans at different temperatures.

### Real space imaging of the VL

**Figure 2:**
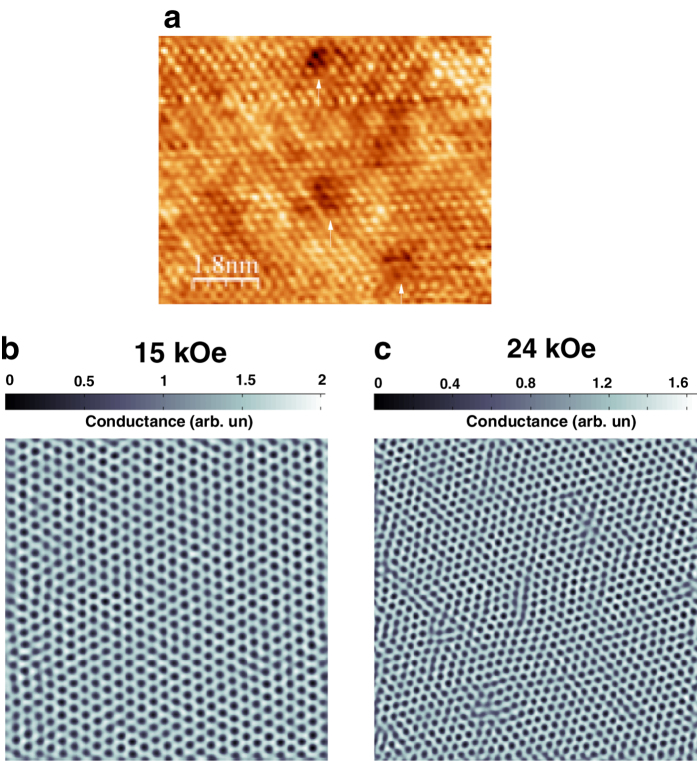
(**a**) Atomic resolution image of the Co_0.0075_NbSe_2_ sample surface. The defects resulting from Co intercalation are visible as darker regions (shown by arrows). (**b**)-(**c**) Representative STS conductance maps on NbSe_2_ over 1 μm × 1 μm area at 15 kOe and 24 kOe. The conductance maps are recorded at d.c. bias voltage of 1.2 mV. The vortices are observed as local minima in the conductance map.

**Figure 3:**
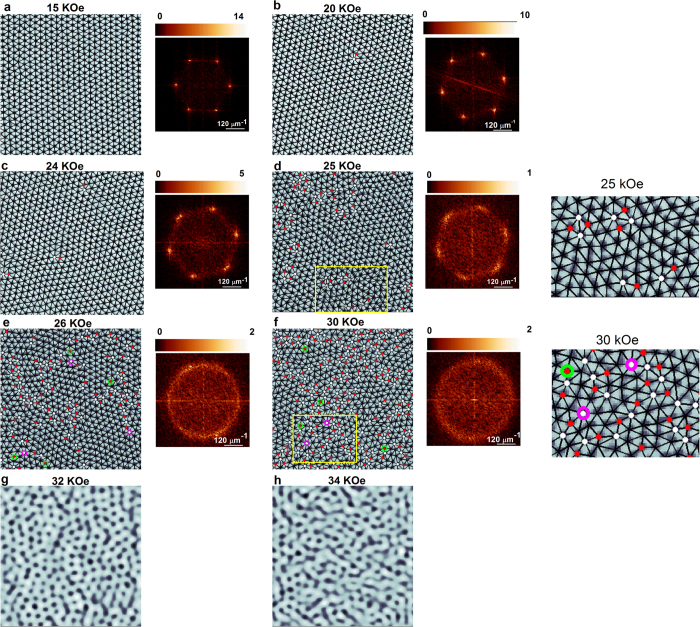
(**a**)-(**f**) STS conductance maps showing real space ZFC vortex lattice image at 350 mK along with their Fourier transforms. Delaunay triangulation of the VL are shown as solid lines joining the vortices and sites with 5-fold and 7-fold coordination are shown as red and white dots respectively. The disclinations (unpaired 5-fold or 7-fold coordination sites) observed at 26 and 30 kOe are highlighted with green and purple circles. While all images are acquired over 1 μm × 1μm area, images shown here have been zoomed to show around 600 vortices for clarity. The Fourier transforms correspond to the unfiltered images; the color scales are in arbitrary units. The expanded view of the defect structure inside the region bounded by the yellow boxes are shown for the VL at 25 and 30 kOe next to panels (**d**) and (**f**) respectively. The images between 20-30 kOe were recorded in the same magnetic field ramp, whereas the image at 15 kOe was recorded in a separate magnetic field ramp on the same cleaved surface. The mis-orientation of the VL at 15 kOe with respect to the other images is due to a small rotation of the sample holder due to vibration during the liquid ^3^He regeneration process between two magnetic field ramps. (**g**)-(**h**) VL images (400 nm × 400 nm) at 32 kOe and 34 kOe. At these fields the VL image becomes blurred due to the motion of vortices.

The VL is imaged using STS over a 1 μm × 1 μm area close to the center of the cleaved crystal surface. Figure 2(a) shows a representative atomic resolution topographic image of the sample surface, where crystalline defects due to Co atoms are seen as dark patches. We first focus on the VL along the ZFC branch. Figure 2(b)-(c) shows the representative conductance maps over the full scan area (1 μm × 1 μm) at 15 kOe and 24 kOe where vortices manifest as a local minima in the conductance. Figure 3 (a)-(f) show the conductance maps superposed with the Delaunay triangulated VL for 6 representative fields, when the magnetic field is ramped up at 350 mK in the ZFC state. The Fourier transforms (FT) corresponding to the unfiltered images are also shown. We identify 3 distinct regimes. For *H* < 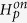, the VL is free from topological defects and the FT show 6 bright spots characteristic of a hexagonal lattice. Between 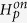 < *H* ≤ *H*_*p*_ dislocations (pairs of nearest neighbor lattice points with 5-fold and 7-fold coordination) gradually proliferate in the system. We call this state an orientational glass (OG). The dislocations do not completely destroy the orientational order which can be seen from FTs which continue to display a six-fold symmetry. For *H* *>* *H*_*p*_ the disclinations (isolated lattice points with 5-fold or 7-seven fold coordination) proliferate in the system driving the VL into an isotropic VG. The FT shows a ring, characteristic of an isotropic disordered state. We observe a significant range of phase coexistence^36^, where both large patches with dislocations coexist with isolated disclinations. Going to higher fields, 32 and 34 kOe (Fig. 3 (g)-(h)) we observe that the VL gets gradually blurred to form a randomly oriented linear structures, where the vortex lines start moving along preferred directions determined by the local surrounding. This indicates a softening of the VG and a gradual evolution towards the liquid state close to *H*_*c2*_.

Further quantitative information on this sequence of disordering is obtained from the orientational and positional correlation functions, 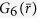 and 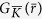, which measure the degree of misalignment of the lattice vectors and the relative displacement between two vortices separated by distance *r* respectively, with respect to the lattice vectors of an ideal hexagonal lattice. The orientational correlation function is defined as^32^, 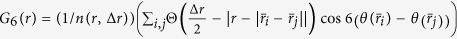, where 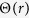 is the Heaviside step function, 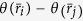 is the angle between the bonds located at 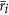 and the bond located at 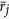, 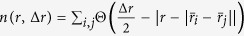, 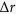 defines a small window of the size of the pixel around *r* and the sums run over all the bonds. We define the position of each bond as the coordinate of the mid-point of the bond. Similarly, the spatial correlation function, 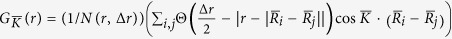, where ***K*** is the reciprocal lattice vector obtained from the Fourier transform, *R*_*i*_ is the position of the *i*-th vortex, 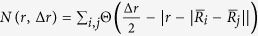 and the sum runs over all lattice points. We restrict the range of *r* to half the lateral size (1 μm) of each image, which corresponds to 11a_0_ (where a_0_ is the average lattice constant) at 10 kOe and 17a_0_ for 30 kOe. For an ideal hexagonal lattice, 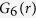 and 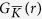 shows sharp peaks with unity amplitude around 1^st^, 2^nd^, 3^rd^ etc… nearest neighbour distance for the bonds and the lattice points respectively. As the lattice disorder increases, the amplitude of the peaks decay with distance and neighbouring peaks at large *r* merge with each other.

**Figure 4:**
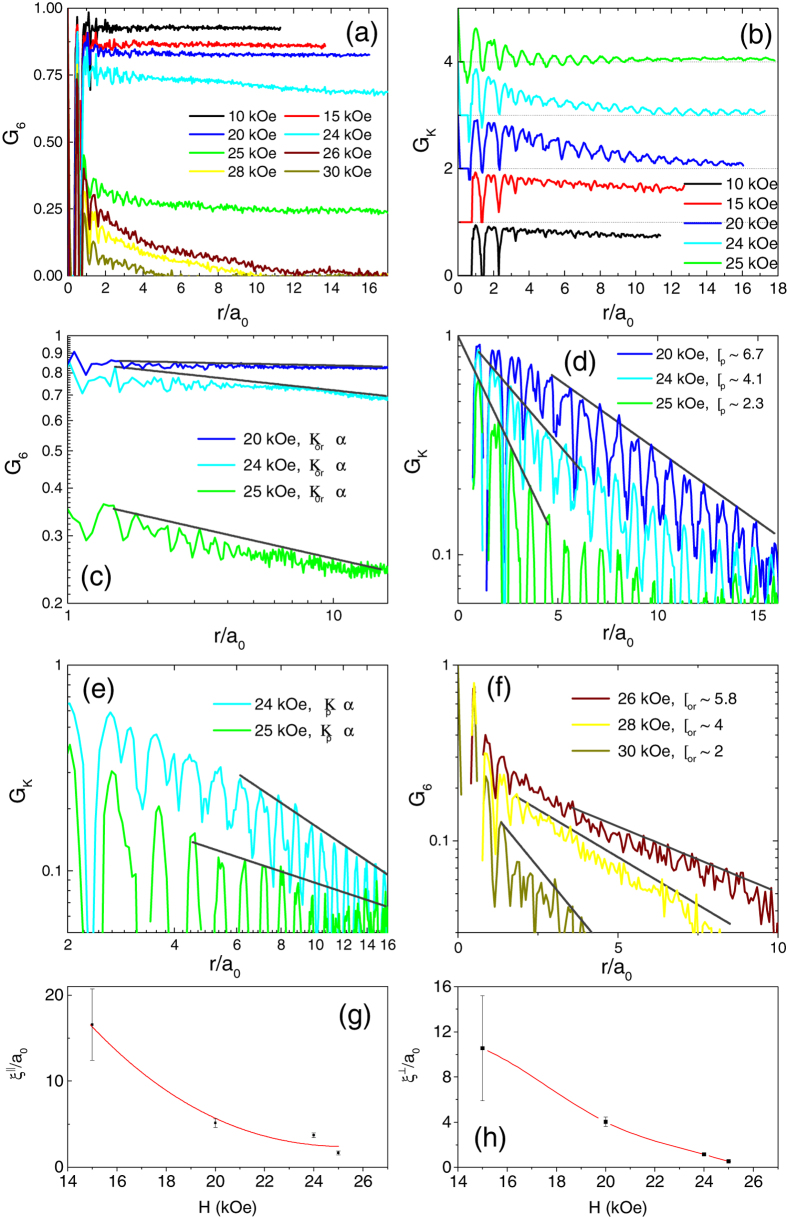
(**a**) Orientational correlation function, G_6_ and (**b**) and positional correlation function, G_K_ (averaged over the principal symmetry directions) as a function of r/a_0_ for the ZFC state at various fields. a_0_ is calculated by averaging over all the bonds after Delaunay triangulating the image. In panel (**b**), G_K_ for each successive fields are separated by adding a multiple of one for clarity. (**c**) G_6_ plotted in log-log scale for 20 kOe, 24 kOe and 25 kOe, along with fits (grey lines) of the power law decay of the envelope; *η*_or_ are the exponents for power-law decay of G_6_. (**d**) G_K_ for 20 kOe, 24 kOe and 25 kOe plotted in semi-log scale along with the fits (grey lines) to the exponential decay of the envelope at short distance, with characteristic decay length, ξ_p_. (**e**) G_K_ for 24 and 25 kOe log-log scale along with fits (grey lines) to the power-law decay of the envelope at large distance; *η*_p_ are the exponents for power-law decay of G_K_. (**f**) G_6_ plotted in semi-log scale for 26 kOe, 28 kOe, 30 kOe along with the fits (grey lines) to the exponential decay of the envelope with characteristic decay length, ξ_or_. (**g**)-(**h**) Variation of the VL correlations lengths 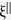 and 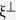 with magnetic field; below 15 kOe the correlation lengths reach the size of the image.

Figure 4(a) and (b) show the 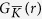 (averaged over the 3 principal ***K*** directions) and 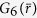, calculated from individual VL images, as a function of *r*/a_0_ for different fields. At 10 kOe and 15 kOe, 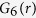 saturates to a constant value of ~0.93 and ~0.86 respectively after 2-3 lattice constants, indicating that the presence of long-range orientational order. The envelope of 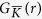 decays slowly but almost linearly with *r.* Since the linear decay cannot continue for large *r*, this reflects our inability to capture the asymptotic behaviour at large *r* at low fields due to limited field of view. While we cannot ascertain whether 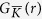 decays as a power-law for large *r* as predicted for a BG, the slow decay of 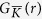 combined with the long-range orientational order is indicative of quasi long-range positional order (QLRPO). In the OG state (20-25 kOe), 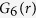 decays slowly with increasing *r*. The decay of *G*_6_(*r*) with *r* is consistent with a power-law 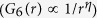, characteristic of quasi-long-range orientational order (Fig. 4(c)). 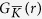, on the other hand displays a more complex behaviour. At 20 kOe, within our field of view the 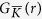 envelope decays exponentially with positional decay length, *ξ*_*p*_ ~ 6.7. However for 24 and 25 kOe where the initial decay is faster, we observe that the exponential decay is actually restricted to small values of *r*/a_0_ (Fig. 4(d)), whereas at higher values 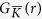 decays as a power-law (Fig. 4(e)). The OG state thus differs from the QLRPO state in that it does not have a true long-range orientational order. It also differs from the hexatic state in 2-D systems, where 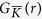 is expected to decay exponentially at large distance. Similar variation of 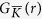 has earlier been reported at intermediate fields in the VL of a neutron irradiated NbSe_2_ single crystal^32^, although the data in that case did not extend to the VG state. Finally, above 26 kOe, 
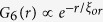
 (*ξ*_*or*_ is the decay length of orientational order), giving rise to regular amorphous VG state with short-range positional and orientational order (Fig. 4(f)).

The possibility of a state with hexatic correlations between the onset and the peak of the “peak effect” has earlier been suggested from the field variation of the positional correlation length of the VL parallel (ξ^||^) and perpendicular 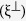 to the reciprocal lattice vector ***K,*** from neutron scattering studies in Nb single crystal^37^. In that measurement, ξ^||^ and 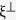 was inferred from the radial width and azimuthal width of the six first order scattering in the Ewald sphere, projected on the plane of the detector^38^. While the relation between these correlation length and the ones obtained from the decay of 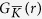 or 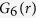 is not straightforward, it is nevertheless instructive to compare the corresponding correlation lengths obtained from our data. We obtain the corresponding lengths in our experiment from the average radial (Δ*k*_*||*_) and azimuthal 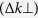 width of the six first-order Bragg peaks of the reciprocal lattice of each VL, obtained by taking the Fourier transform of a binary map constructed using the position of the vortices (See Methods and Supplementary material). Since the Bragg peaks fit well to a Lorentzian profile, we correct for the peak broadening arising from the finite size of the images and the position uncertainly arising from the finite pixel size of the images by subtracting the peak width of the Bragg spots of an ideal hexagonal lattice of the same size constructed on a grid containing the same number of pixels. Figure 4 (g)-(h) show the variation of 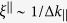 and 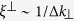 with magnetic field. We observe that 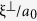 and 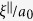 decreases rapidly between 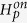 and *H*_*p*_ signalling the progressive decrease in orientational and positional order. At *H*_*p*_, both 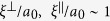, showing both positional and orientational order are completely lost. Both the magnitude and field dependence of 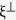 and 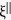 are qualitatively consistent with ref. 37 even though the measurements here are performed at a much lower temperature.

**Figure 5:**
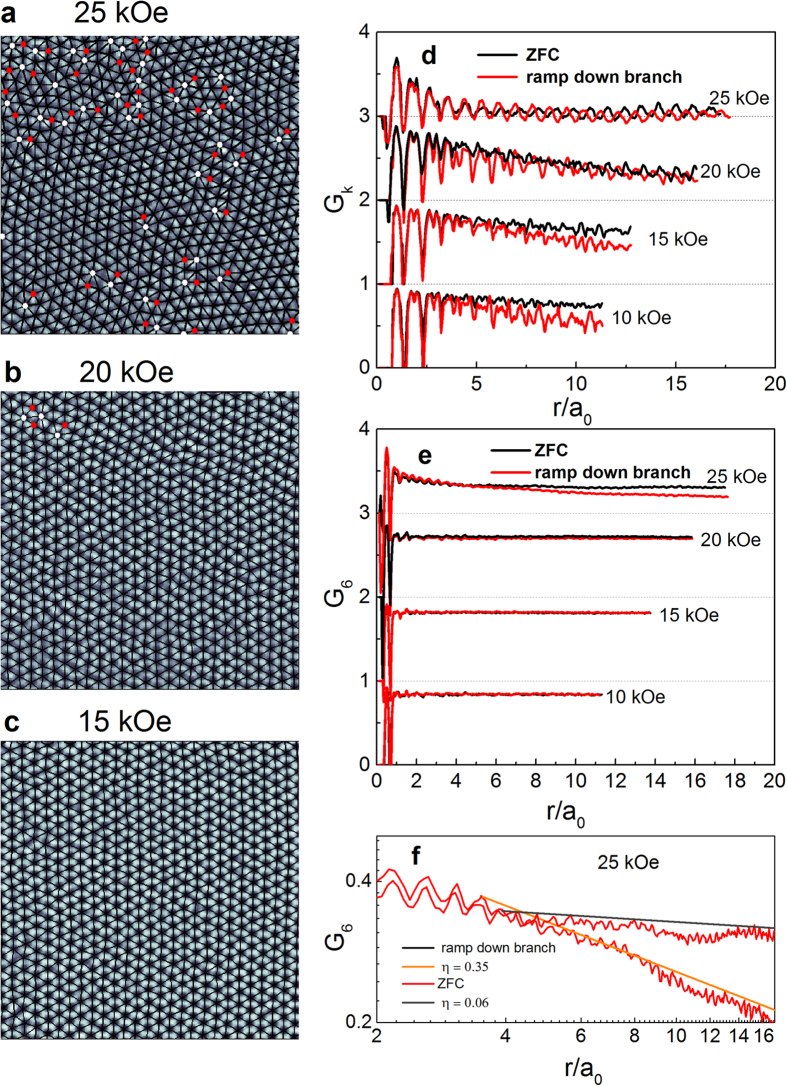
(**a**)-(**c**) VL images at 350 mK along with Delaunay triangulation for the ramp down branch at 25, 20 and 15 kOe. Sites with 5-fold and 7-fold coordination are shown as red and white dots respectively. At 25 and 20 kOe we observe dislocations in the VL. At 15 kOe the VL is topologically ordered. While all images are acquired over 1 μm × 1μm area, images shown here have been zoomed to show around 600 vortices for clarity. (**d**) G_6_ and (**e**) G_**K**_ calculated from the VL images at different field when the magnetic field is ramped down from *H* *>* *H*_*c2*_at 350 mK. The curves for each successive field are separated by adding a multiple of one for clarity. (**f**) G_6_ at 25 kOe for ZFC and ramp down branch along with the fit (solid lines) to the power-law decay of the corresponding envelope; the exponents for the power-law fits, *η*, are shown in the legend.

We now discuss the ramp down branch focussing on the hysteresis region in the χ’−*H* measurements. Figure 5(a)-(c) shows the VL configurations for the ramp down branch at 25, 20 and 15 kOe. The VL structures for the ramp down branch are similar to ZFC: At 25 and 20 kOe the VL shows the presence of dislocations and at 15 kOe it is topologically ordered. In Fig. 4(d)-(f) we compare 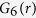 and 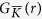 calculated for the ZFC and the ramp down branch. At 25 kOe ≈ *H*_*p*_, we observe that 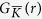 for ZFC and ramp down branch are similar whereas 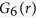 decays faster for the ramp down branch. However, analysis of the data shows that in both cases 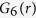 decays as a power-law (Fig. 5(f)) characteristic of the OG state. At 15 kOe, which is just below 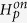, both ZFC and ramp down branch show long-range orientational order, while 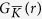 decays marginally faster for the ramp down branch. Thus, while the VL in the ramp down branch is more disordered, our data do not provide any evidence of supercooling across either QLRPO → OG or OG → VG transitions as expected for a first order phase transition. Therefore, we attribute the hysteresis to the inability of the VL to fully relax below the transition in the ramp down branch.

**Figure 6:**
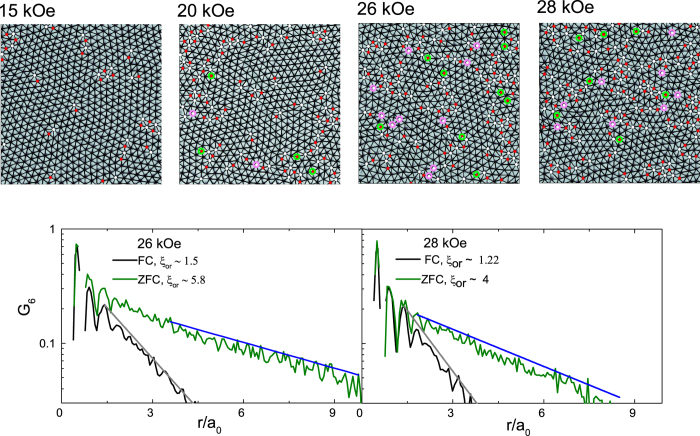
Field Cooled VL images (upper panels) at 15 kOe, 20 kOe, 26 kOe, 28 kOe at 350 mK. Delaunay triangulation of the VL are shown as solid lines joining the vortices and sites with 5-fold and 7-fold coordination are shown as red and white dots respectively. The disclinations are highlighted with green and purple circles. The lower panels show the variation of G_6_ for the FC and ZFC states at 26 kOe and 28 kOe along with the corresponding fits for exponential decay (solid lines); the decay lengths for the orientational order, ξ_or_ are shown in the legend. Images have been zoomed to show around 600 vortices for clarity.

**Figure 7:**
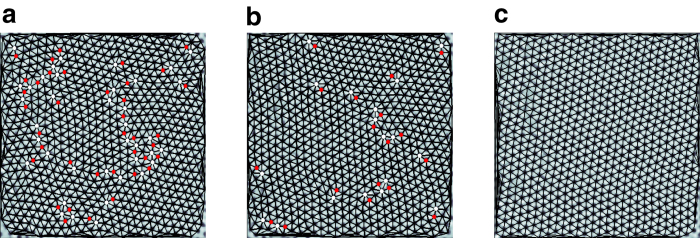
Annihilation of dislocations in a FC vortex lattice at 15 kOe with application of magnetic field pulse. (**a**) FC VL at 350 mK; the same VL after a applying a magnetic field pulse of (**b**) 0.3 kOe and (**c**) 0.9 kOe. Dislocations in the VL are shown as pairs of adjacent points with five-fold (red) and seven-fold (white) coordination. In (**b**) many of the dislocations are annihilated, whereas in (**c**) all dislocations are annihilated. Delaunay triangulation of the VL are shown as solid lines joining the vortices. Images have been zoomed to show around 600 vortices for clarity.

We can now follow the magnetic field evolution of the FC state (Fig. 6). The FC state show an OG at 10 kOe (not shown) and 15 kOe (free dislocations), and a VG above 20 kOe (free disclinations). The FC OG state is however extremely unstable. This is readily seen by applying a small magnetic pulse (by ramping up the field by a small amount and ramping back), which annihilates the dislocations in the FC OG (Fig. 7) eventually causing a dynamic transition to the QLRPO state. It is interesting to note that metastability of the VL persists above *H*_*p*_ where the ZFC state is a VG. The FC state is more disordered with a faster decay in 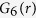 (Fig. 6, lower panels), and consequently is more strongly pinned than the ZFC state.

## Discussion

The sequence of disordering observed in our experiment is reminiscent of the two-step melting observed in 2-D systems^39^, where a hexatic fluid exists as an intermediate state between the solid and the isotropic liquid. However, the situation for the weakly pinned 3-D VL is more complex. Here, the reduced influence of thermal fluctuations prevents from establishing a fluid state in the presence of a pinning potential. Thus, the OG and the VG state are both glassy states with very slow kinetics, which appear completely frozen within the timescales of our experiment, till very close to *H*_*c2*_. The glassy nature of the VL also manifests by producing a number of non-equilibrium states, such as the OG below 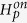 and the VG state below *H*_*p*_ when the sample is field cooled.

One pertinent question in this context is whether the transformation from QLRPO → OG and OG → VG represent continuous phase transitions as in the case of 2-D systems or are simple cross-overs induced by random pinning. Within our definition the OG state is characterized by the presence of dislocations which destroy the long range orientational order. Similarly, in the VG state the proliferation of disclinations destroy the quasi-long range orientational order. However, a finite number of topological defects need not necessarily destroy the positional or orientational order. For example, it has been argued that the QLRPO might survive in the presence of small amounts of dislocations as long as the average distance between dislocations is much larger than the effective correlation length^4^. Therefore, this issue remains unresolved from the analysis of finite size images, and has to be studied through further thermodynamic measurements. However, it is interesting to note that we do not observe any evidence of supercooling across the peak effect as expected for a first-order phase transition. Theoretically the possibility of a second order field induced melting transition has been speculated^40^, though detailed calculations do not exist. Finally, the gradual softening of the VG as the magnetic field approaches *H*_*c2*_ supports the view^13^ that the VG and the vortex liquid are thermodynamically identical states in two different limits of viscosity.

The sequence of disordering observed in our experiment bears some similarity with the phase diagram proposed in ref. 14 where the BG disorders to a VG through an intermediate “multidomain glass” (MG) state. However, in a MG the spatial correlations are similar to a BG up to a characteristic length-scale followed by a more rapid decay at larger distance. In contrast, in the intermediate OG state observed in our experiment, 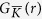 decays rapidly at short distance followed by a more gradual behaviour. The possibility of the BG transforming to an intermediate state that retains hexatic correlations, which would be closer to our experimental findings, has also been proposed as a possibility^14^,^40^, but has not been explored in detail.

One final question is the role of the symmetry of the underlying crystalline lattice on the orientation of the VL. The coupling of the VL with the symmetry of the crystalline lattice has been demonstrated to give rise to non-trivial distortions of the VL when the symmetry of the crystal lattice is not hexagonal^41^. In our case however, NbSe_2_ has a layered hexagonal structure and the magnetic field is applied along the six-fold symmetric *c*-axis. In this case, very recent experiments performed in our laboratory reveals that the VL is preferentially oriented along the crystallographic direction (see supplementary material). It is therefore possible that orientational pinning of the VL to the crystal lattice plays an important role, contributing to the stability of the orientational order in OG state. This has to be explored in detail in future.

In summary, the physical picture emerging from our measurements is that across the peak effect, the VL in a weakly pinned Type II superconductor disorders through gradual destruction of positional and orientational order. Our experiments do not provide evidence of a first order order-disorder transition across the field driven peak effect at low temperatures. In this context we would like to note that a hexatic VL state (similar to the OG state), believed to represent the “ordered state” of vortex matter, has earlier been observed from magnetic decoration experiments on a layered high-*T*_*c*_ Bi_2.1_Sr_1.9_Ca_0.9_Cu_2_O_8+δ_ single crystals at low temperatures and very low fields^42^. It would therefore be interesting to carry out further experiments on other low-*T*_*c*_ and high-*T*_*c*_ superconductor with different levels disorder which would provide valuable insight on the range of parameter space over which the OG state is observed in systems with different disorder and anisotropies.

## Material and Methods

### Sample preparation

The Co_0.0075_NbSe_2_ single crystal was grown by iodine vapour transport method starting with stoichiometric amounts of pure Nb, Se and Co, together with iodine as the transport agent. Stoichiometric amounts of pure Nb, Se and Co, together with iodine as the transport agent were mixed and placed in one end of a quartz tube, which was then evacuated and sealed. The sealed quartz tube was heated up in a two zone furnace for 5 days, with the charge-zone and growth-zone temperatures kept at, 800 °C and 720 °C respectively. We obtained single crystals of nominal composition Co_0.0075_NbSe_2_ with lateral size of 4-5 mm. The crystal on which the measurements were performed had a superconducting transition temperature, *T*_*c*_ ~ 5.3 K with a resistive transition width of 200 mK (see supplementary material) and *H*_*c2*_ ~ 38 kOe (for 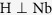 planes). The same sample was used for susceptibility and STS measurements.

### a.c. suspectibility measurements

a.c. susceptibility measurements were performed down to 350 mK using a home-built susceptometer, in a ^3^He cryostat fitted with a superconducting solenoid. Both ac and dc magnetic field were applied perpendicular to the Nb planes. The dc magnetic field was varied between 0-60 kOe. The ac excitation field was kept at 10 mOe where the susceptibility is in the linear response regime (see supplementary material).

### Scanning tunneling spectroscopy measurements.

The VL was imaged using a home-built scanning tunneling microscope^43^ (STM) operating down to 350 mK and fitted with an axial 90 kOe superconducting solenoid. Prior to STM measurements, the crystal is cleaved *in-situ* in vacuum, giving atomically smooth facets larger than 1 μm × 1 μm. Well resolved images of the VL are obtained by measuring the tunneling conductance (*G(V) *= *dI/dV*) over the surface at a fixed bias voltage (*V* ~ 1.2 mV) close to the superconducting energy gap, such that each vortex core manifests as a local minimum in *G(V)*. Each image was acquired over 75 minutes after waiting for 15 minutes after stabilizing to the magnetic field. The precise position of the vortices are obtained from the images after digitally removing scan lines (see supplementary material) and finding the local minima in *G(V)* using WSxM software^44^. To identify topological defects, we Delaunay triangulated the VL and determined the nearest neighbor coordination for each flux lines. Topological defects in the hexagonal lattice manifest as points with 5-fold or 7-fold coordination number. Since, the Delaunay triangulation procedure gives some spurious bonds at the edge of the image we ignore the edge bonds while calculating the average lattice constants and identifying the topological defects. Unless otherwise mentioned VL images are taken over an area of 1 μm × 1 μm. The correlation functions G_6_ and G_K_ are calculated using individual images.

## Additional Information

**How to cite this article**: Chandra Ganguli, S. *et al.* Disordering of the vortex lattice through successive destruction of positional and orientational order in a weakly pinned Co_0.0075_NbSe_2_ single crystal. *Sci. Rep.* **5**, 10613; doi: 10.1038/srep10613 (2015).

## Supplementary Material

Supplementary Information
