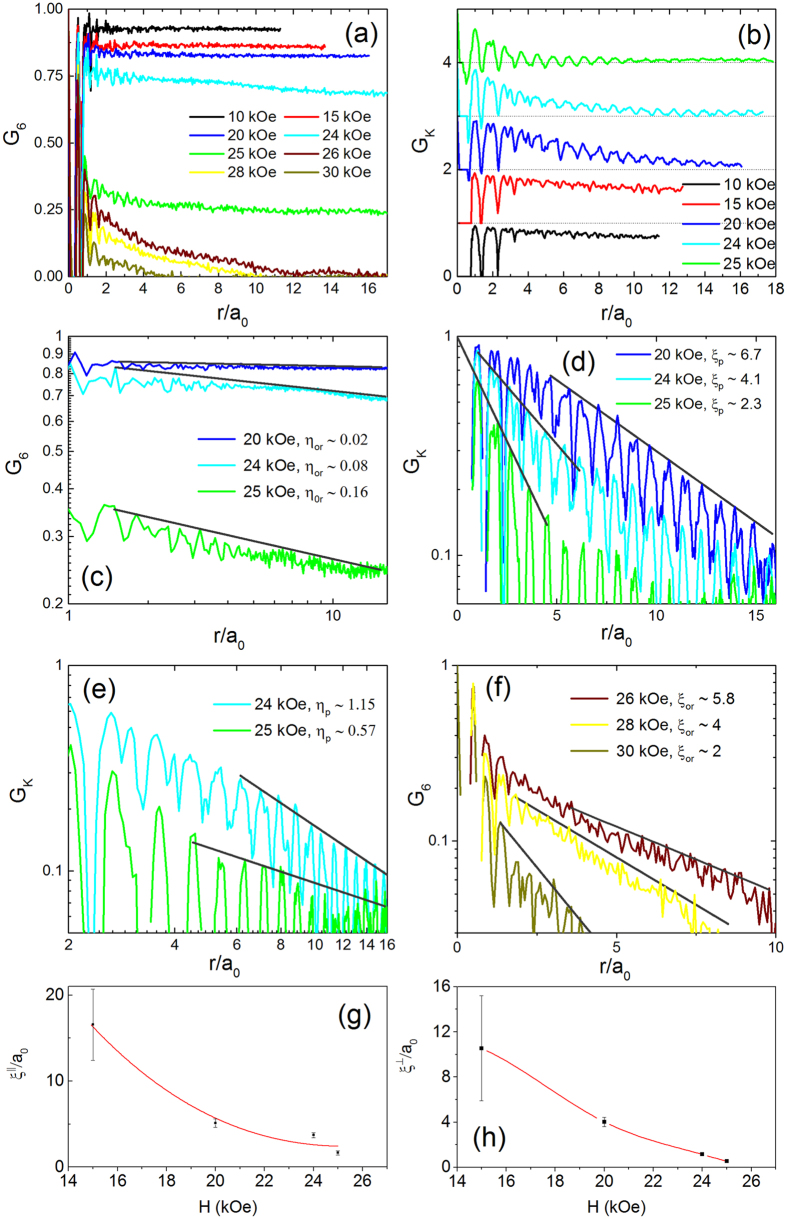# Erratum: Disordering of the vortex lattice through successive destruction of positional and orientational order in a weakly pinned Co_0.0075_NbSe_2_ single crystal

**DOI:** 10.1038/srep17923

**Published:** 2016-01-12

**Authors:** Somesh Chandra Ganguli, Harkirat Singh, Garima Saraswat, Rini Ganguly, Vivas Bagwe, Parasharam Shirage, Arumugam Thamizhavel, Pratap Raychaudhuri

Scientific Reports 5: Article number: 1061310.1038/srep10613; published online: 06032015; updated: 01122016

This Article contains typographical errors in the keys of Figure 4c, 4d, 4e, and 4f.

In Figure 4c, the values of the exponents for power-law decay of G_6_ ‘*η*_or_’ have been incorrectly given as ‘α’.

In Figure 4e the values of the exponents for power-law decay of G_K_ ‘*η*_p_’ have been incorrectly given as ‘α’.

Lastly in Figures 4d and 4f, there are typographical errors in the symbols ‘ξ_p_’ and ‘ξ_or_’ which were incorrectly given as ‘[_p_’ and ‘[_or_’ respectively.

The correct Figure 4 appears below as Fig. 1.

## Figures and Tables

**Figure 1:**