# Supplementary material for: Disordering of the vortex lattice through successive destruction of positional and orientational order in a weakly pinned Co0.0075NbSe2 single crystal
Source: Sci Rep. 2015 Jun 3;5:10613. doi: 10.1038/srep10613 (PMC4454145; doi:10.1038/srep10613)
Supplement: Supplementary Information [file srep10613-s1.pdf]

Disordering of the vortex lattice through successive destruction of positional and orientational order in a weakly pinned $Co_{0.0075}NbSe_2$ single crystal

Somesh Chandra Ganguli[a], Harkirat Singh[a], Garima Saraswat[a], Rini Ganguly[a], Vivas Bagwe[a], Parasharam Shirage[b], Arumugam Thamizhavel[a] and Pratap Raychaudhuri[a1]

[a] *Tata Institute of Fundamental Research, Homi Bhabha Road, Colaba, Mumbai 400005, India.*

[b] *Indian Institute of Technology Indore, IET-DAVV Campus, Khandwa Road, Indore 452017, India.*

I. Temperature dependence of resistance of $Co_{0.0075}NbSe_2$ single crystal

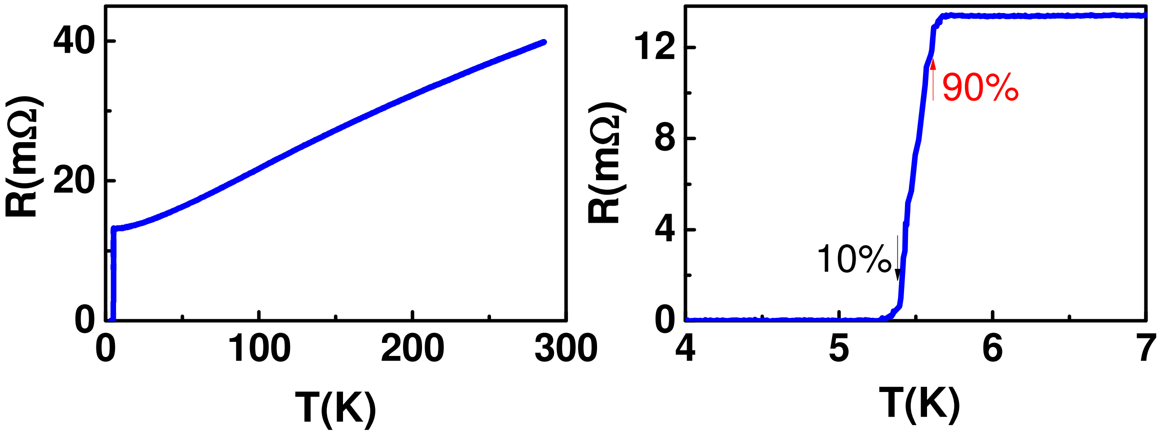

Fig S1| Resistance vs. temperature for the $Co_{0.0075}NbSe_2$ sample. The left panel shows the resistivity from 286 - 4 K. The right panel shows an expanded view close to the superconducting transition. The arrows mark the positions where the resistance is 90% and 10% of the normal state value respectively.

The $Co_{0.0075}NbSe_2$ single crystal was characterised using 4-probe resistivity measurements from 286 K to 4 K (Fig. S1). The superconducting transition temperature, defined as the temperature where the resistance goes below our measurable limit is 5.3 K. The transition width, defined as the difference between temperatures where the resistance is 90% and 10% of the normal state resistance respectively is ~ 200 mK. The same crystal was used for both a.c. susceptibility and STS measurements.

[1] E-mail: pratap@tifr.res.in

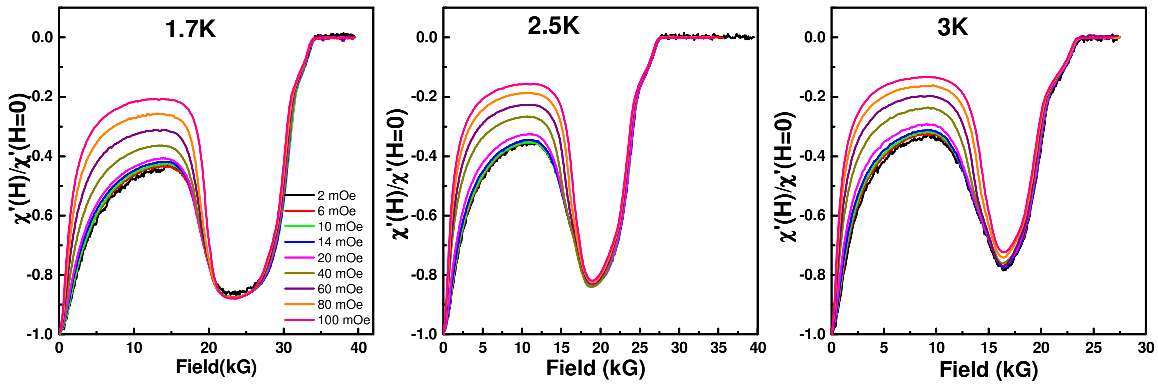

Fig S2| Isothermal χ'-H scans for different a.c. excitations. The susceptibility response shows a strong amplitude dependence above 14 mOe.

II. a.c. susceptibility response as a function of excitation field

The a.c. susceptibility reported in Fig. 1 was performed with an a.c. excitation amplitude of 10 mOe at frequency 31 kHz. Since at large amplitudes, the a.c. drive can significantly modify the susceptibility response of the VL through large scale rearrangement of vortices, we performed several measurements with different a.c. excitation amplitudes to determine the range of a.c. field over which the χ' is independent of excitation field. We observe (Fig. S2) that below 3K, χ' shows significant dependence on the magnitude of the a.c. excitation only above 14 mOe.

III. Calculation of the correlation lengths $\xi^{\|}$ and $\xi^{\perp}$ from VL images

For an infinite lattice the correlation lengths along and perpendicular to the reciprocal lattice vectors **K** can be obtained from the width of the first order Bragg peaks (BP) of the reciprocal lattice using the relations, $\xi^{\|} = 1/\Delta k_{\|}$ and $\xi^{\perp} = 1/\Delta k_{\perp}$, where $\Delta k_{\|}$ and $\Delta k_{\perp}$ are the width of the first order Bragg peaks parallel and perpendicular to **K**. This method has been used to determine the correlation lengths from the Bragg spots in neutron diffraction measurements. However, when using the Fourier transform of VL images obtained from STS measurements, additional precaution has to be taken to account for contributions arising from the finite size of images and any inaccuracy in position resulting from the finite pixel resolution.

To obtain $\xi^{\parallel}$ and $\xi^{\perp}$ from our data, at every field a binary lattice is first constructed using the position of each vortex (Fig. S3(a)) obtained the VL images as explained in the Methods section.

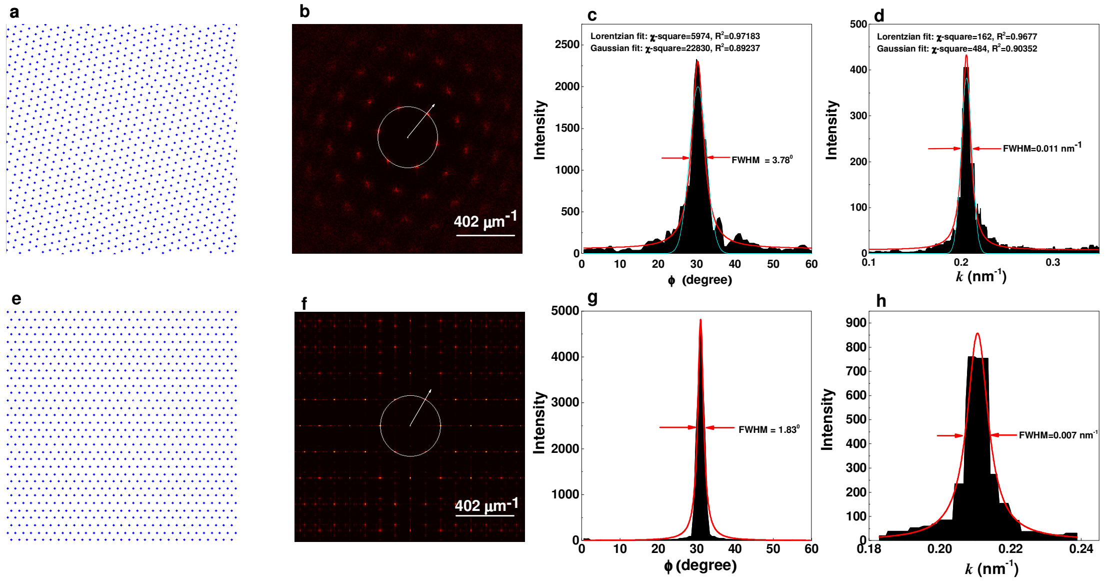

Fig. S3| (a) Binary image of the vortex positions constructed from the VL image at 20 kOe. (b) 2D Fourier transform of (a) showing one reciprocal lattice vector, **K**. (c) Azimuthal profile of the first order Bragg peak averaged over the six symmetric peak along the circle shown in (b); ϕ is the azimuthal angle with respect to an arbitrary axis roughly midway between two Bragg peaks. (d) Radial profile of the First order Bragg peak along the reciprocal lattice vector averaged over the six symmetric peaks; $k_{||}$ is the magnitude of wave-vector along the reciprocal lattice vector. The lines show the fit to the Lorentzian (red) and the Gaussian (indigo) functions; the corresponding χ-square and R^2 values are shown in the panel. (e)-(h) Same as (a)-(d) for the ideal hexagonal lattice of same size with the same density of lattice points.

This removes image specific features and leaves only the precise position of each vortex within the uncertainty of pixel resolution. The 2D Fourier transform of this image reveals the first order Bragg peaks which appear as six symmetric bright spots as well as higher order peaks (Fig. S3(b)). (The position of the first order peaks with respect to the origin correspond to the reciprocal lattice vector, **K**.) We determine the peak profile along the azimuthal direction by taking the cut along a circle going through the center of the six first order Bragg spots. Similarly, the profile along the radial direction is determined by taking a line cut along the direction of the reciprocal lattice vector. We tried to fit peak profile averaged over the six symmetric BPs with both Lorentzian and Gaussian functions. The goodness of fit can be estimated from the residual sum of squares (χ-square) and the coefficient of determination[1] (adjusted R^2) which should be 1 when the fit is perfect. We observe that the Lorentzian function gives a much better fit with smaller χ-square and R^2 value close to unity. Therefore

we calculate the corresponding peak width from the full width at half maxima of the best fit Lorentzian function (Fig. S3(c)-(d)).

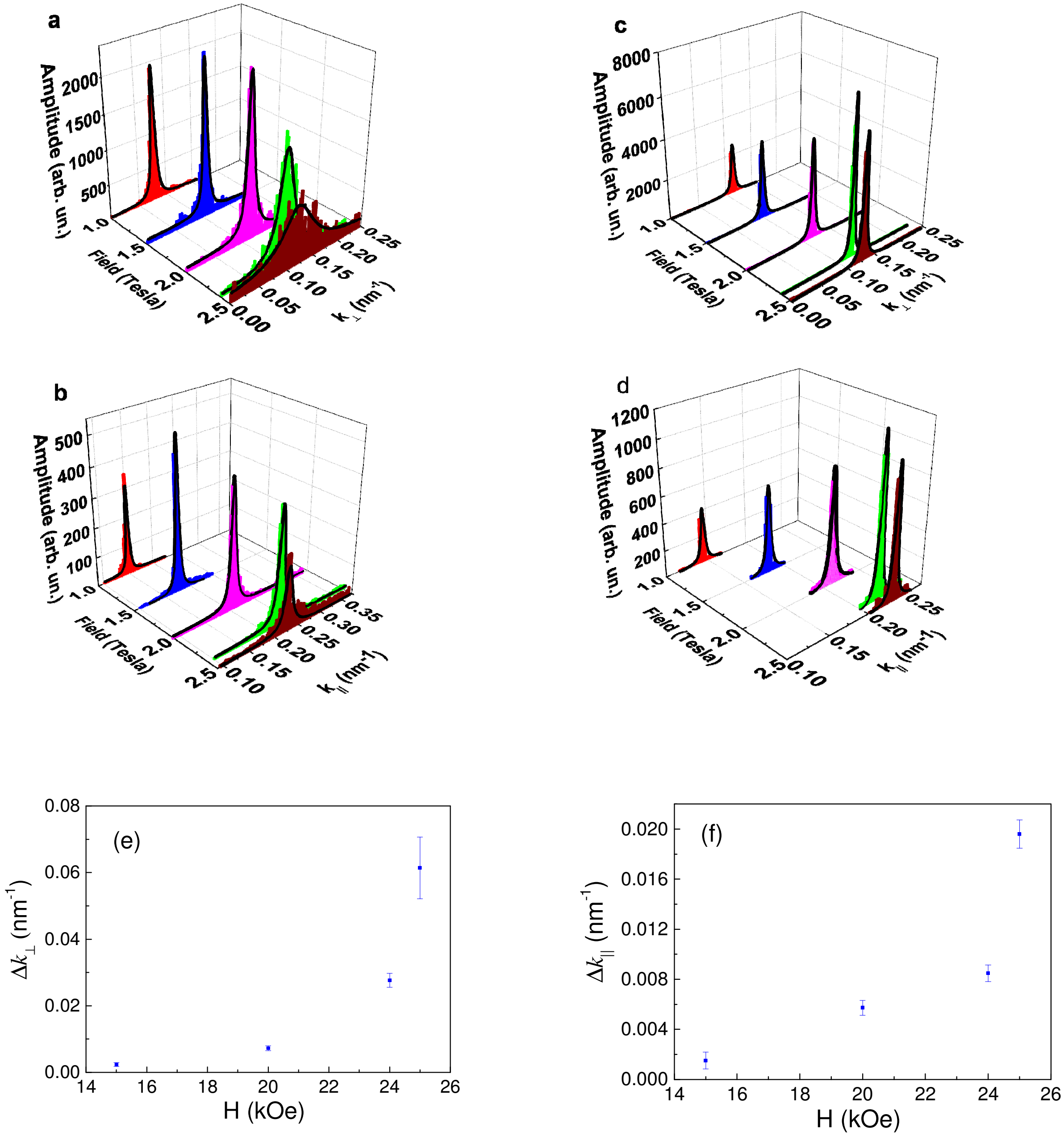

Fig. S4| (a)-(b) Profiles of the first order Bragg peaks (averaged over the six symmetric peaks) along the radial and azimuthal directions at different fields obtained from the VL images shown in Fig. 2 of the main paper. $k_\perp = \phi_0$ (in radian) × |**K**| is the distance in reciprocal space measured along the azimuthal direction. (c)-(d) Corresponding peak profiles for an ideal hexagonal lattice of the same size and same density of lattice points. The black lines are the fits to a Lorentzian function. (e)-(f) Variation of $\Delta k_\perp$ and $\Delta k_{||}$ with magnetic field.

Three factors contribute to the peak width determined in this process: (i) The intrinsic disorder in the lattice, (ii) the finite size of the image and (iii) the positional uncertainty arising from the finite pixel resolution of our images. For each field we construct the binary image of an ideal hexagonal lattice of the same size with the same density of lattice points, where the position of each lattice point is rounded off to same accuracy as the pixel resolution of our image (256 × 256). The radial and the azimuthal width of the first order Bragg spots for this ideal lattice is determined using the same procedure as before (see Fig. S3(e)-(h)). In principle the experimental peak width is a convolution of intrinsic and extrinsic factors, and to correct for the contributions arising from extrinsic factors one needs to follow an elaborate deconvolution procedure. However, when the peak can be fitted with a pure Lorentzian function, the situation is simpler and the peak widths arising from different contributions are additive. Since in our case we can fit the Bragg peaks with a pure Lorentzian function, we subtract the peak width of the ideal lattice from the peak width obtained from the actual image, to obtain $\Delta k_{\perp}$ and $\Delta k_{||}$ arising from the lattice disorder alone.

Fig. S4 shows the evolution of azimuthal and radial peak profile averaged over the six first order Bragg spots as a function of magnetic field for the actual (Fig. S4(a)-(b)) and the ideal lattice (Fig. S4(c)-(d)). Fig. S4(e)-(f) show the magnetic field variation of the $\Delta k_{\perp}$ and $\Delta k_{||}$ extracted from this data. We note that the evaluation of the correlations lengths from the relations, $\xi^{||} = 1/\Delta k_{||}$ and $\xi^{\perp} = 1/\Delta k_{\perp}$ is only valid up to the size of the image. Consequently, for our data $\xi^{||}$ and $\xi^{\perp}$ are evaluated only for fields of 15 kOe and higher.

IV. Filtering the STS conductance maps

For better visual depiction, the conductance maps obtained from STS are digitally filtered to remove the noise and scan lines which arise from the raster motion of the tip. The filtering procedure is depicted in Fig. S5. Fig. S5(a) shows the raw conductance map obtained at 24 kOe. To filter the image we first obtain the 2D Fourier transform (FT) of the image Fig. S5(b). In addition to six bright spots corresponding to the Bragg peaks we observe a diffuse intensity at small $\boldsymbol{k}$ corresponding to the random noise and a horizontal line corresponding to the scan lines. We first remove the noise and scan line contribution from the FT by suppressing the intensity along the horizontal line and the diffuse intensity within a circle at small $\boldsymbol{k}$ (Fig. S5(c)). The filtered image shown in Fig. S5(d) is obtained by taking a reverse FT Fig. S5(c).

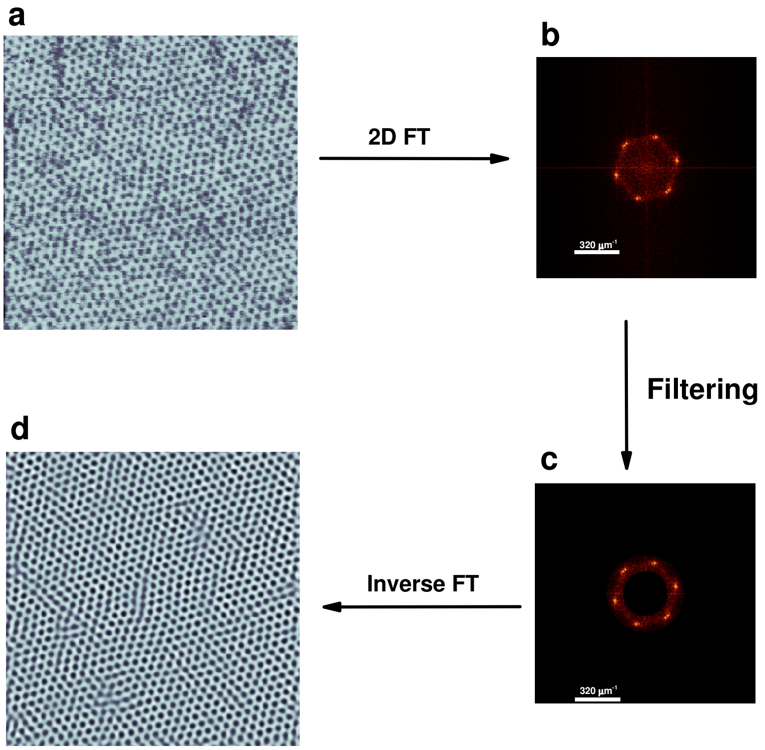

Fig. S5| Filtering the conductance map. (a) Raw conductance map recorded at 24 kOe, 350 mK over an area of 1 µm × 1 µm; (b) 2D FT of (a); (c) 2D FT after removing the contribution from random noise at small ***k*** and scan lines; (d) Filtered image obtained from the inverse FT of (c).

V. Determining the position of the vortices and Delaunay triangulation

To identify topological defects in the VL we need to determine the nearest neighbor coordination of each vortex. For this first the position of individual vortices are obtained from the local minima of the filtered image, generating a map of the center of each vortex (Fig. S6(a)-(b)). The position of each vortex is thus determined within the resolution of 1 pixel which for our images (256 × 256 pixel) is ~ 3.9 nm. Subsequently, the center map is Delaunay triangulated (Fig. S6(c)-(d)) such that there are no points inside the circumcircle of each triangle. This process generates a unique set of bonds connecting the center of vortices. Topological defects are identified from lattice points that are connected to smaller or larger than 6 nearest neighbors (normally 5 and 7 respectively).

An artifact of the Delaunay triangulation process is to generate some spurious bonds at the edge of the image as seen in Fig. S6(c). Thus in our analysis, we ignore all the bonds that are at the edge of the image.

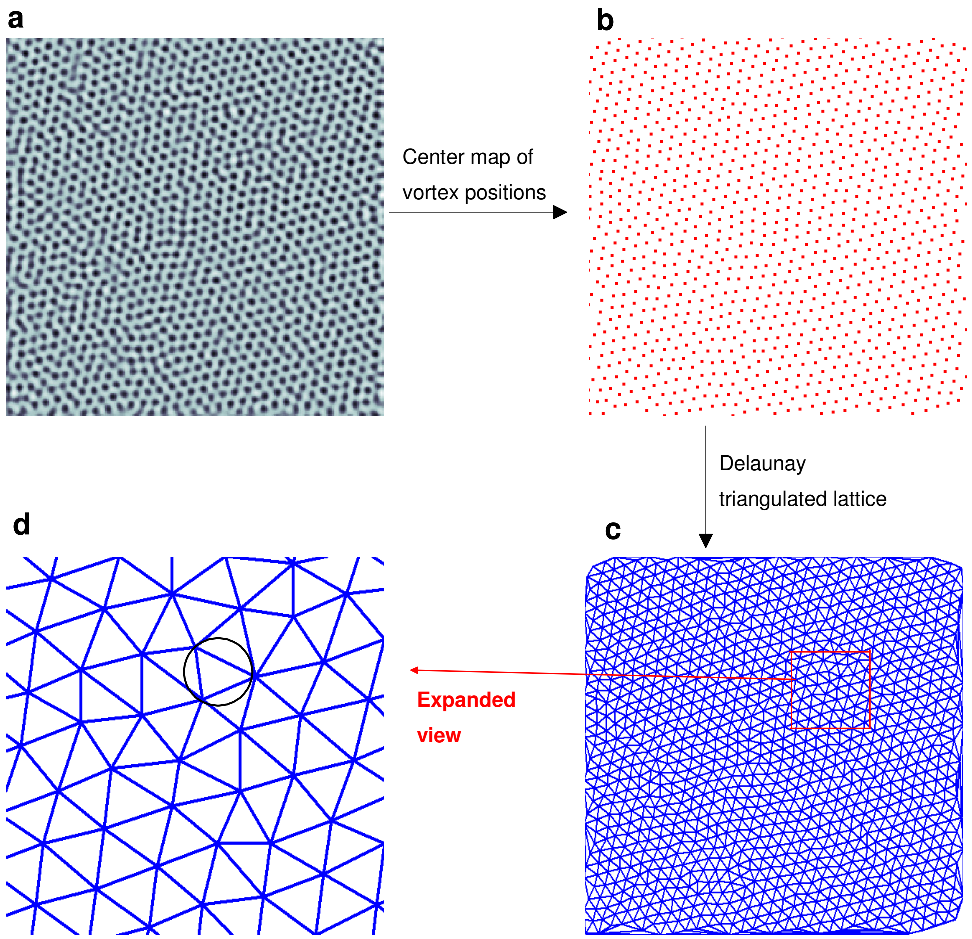

Fig. S6| Delaunay triangulation of the VL. (a) Filtered conductance map showing the ZFC VL at 24 kOe, 350 mK; (b) position of the center of each vortex obtained from the local minima of the conductance map; the size of the red dots is adjusted to reflect the uncertainty is position resulting for finite pixel resolution of our image. (c) Delaunay triangulation of the center map; (d) Expanded view of the Delaunay triangulated lattice showing a representative circumcircle enclosing a triangle.

VI. Orientation of the crystal lattice and VL

The VL can be influenced by the underlying symmetry of the crystal lattice. In our experiment the magnetic field is applied along the six-fold symmetric *c*-axis of the $NbSe_2$ single crystal. By simultaneously imaging the crystalline lattice and the vortex lattice, we observe that the hexagonal vortex lattice gets preferentially oriented along the crystalline axes in the *a*-*b* plane (Fig. S7). This measurement was done on a different Co-doped single crystal from the one reported in the main paper with a higher $T_c \sim 5.9$ K and higher H_p^{on} and H_p.

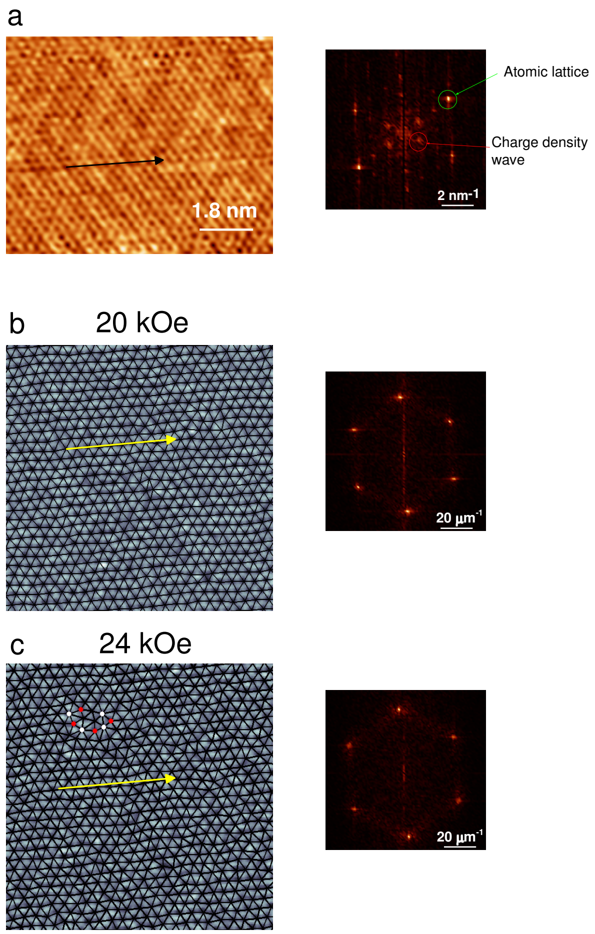

Fig. S7| Orientation of VL and the crystal lattice. (a) Atomic resolution image of the Co doped $NbSe_2$ single crystal showing Se atoms, imaged close to the center of the area over which the VL is imaged. The arrow points towards the direction of one of the hexagonal lattice vector in the *a-b* plane. The corresponding Fourier transform shows sharp six spots corresponding to the atomic lattice and six diffuse spots corresponding the charge density wave modulation. (b)-(c) The vortex lattice imaged at 350 mK (showing approximately 600 vortices) at 20 kOe and 24 kOe. The arrows point towards the direction of one of the lattice vectors of the hexagonal VL. The corresponding Fourier transforms are shown next to each images. Both from the real space image and the Fourier transform it is seen that the VL is oriented along the crystalline lattice.

---

[1] Glantz, Stanton A.; Slinker, B. K. (1990). Primer of Applied Regression and Analysis of Variance. McGraw-Hill. ISBN 0-07-023407-8.
